# Effect of Al Content on the Microstructure and Corrosion Resistance of Low-Pressure Cold-Sprayed Fe-Al Coatings

**DOI:** 10.3390/ma19091852

**Published:** 2026-04-30

**Authors:** Yafei Liu, Zhi Jia, Yanqin Zhang

**Affiliations:** 1 State Key Laboratory of Advanced Processing and Recycling of Nonferrous Metals, Lanzhou University of Technology, Lanzhou 730050, China; 15137280429@163.com (Y.L.); 19029339565@163.com (Y.Z.); 2 School of Material Science and Engineering, Lanzhou University of Technology, Lanzhou 730050, China

**Keywords:** low-pressure cold spraying, Fe-Al composite coating, 45 steel, corrosion resistance, sacrificial anode protection

## Abstract

Using low-pressure cold spray technique, Fe-Al composite coatings with different Al contents were applied to the surface of 45 steel to improve its corrosion resistance in chloride-containing settings. The microstructure, mechanical characteristics, and electrochemical corrosion behavior of the coatings were thoroughly examined in relation to the Al content (2, 4, 6, and 8 wt.%). The findings show that the microhardness of the composite coating decreases monotonically (from 157.98 HV to 99.29 HV) as the Al content rises because of the increased proportion of the soft phase; in contrast, the porosity and corrosion current density show a pattern of first decreasing and then increasing. The coating porosity was reduced to a minimum (1.37%) when the Al concentration reached 6 wt.% because the soft Al particles experienced enough plastic flow to fill the holes in the hard Fe matrix. The 6Al composite coating demonstrated the best electrochemical protection performance in a 3.5 wt.% NaCl solution, with the lowest corrosion current density (2.237 × 10^−4^ A/cm^2^) and the strongest interfacial charge transfer resistance. The synergistic corrosion protection mechanism comprising significantly densified physical shielding and microgalvanic sacrificial anode protection by the active Al phase was clarified in this study. The ideal composition ratio for this system was determined to be 6 wt.% Al by carefully matching the coating’s mechanical load-bearing needs with long-term corrosion prevention goals.

## 1. Introduction

Because of its superior overall mechanical qualities and inexpensive processing, 45 steel is a premium carbon structural steel that is extensively utilized in the mechanical manufacturing sector. However, 45 steel’s surface is extremely vulnerable to electrochemical corrosion in harsh service environments including chloride ions because of its moderately reactive chemical characteristics, which reduces the equipment’s service life. Therefore, improving the corrosion resistance and service life of 45 steel can be achieved by implementing effective surface protection strategies while preserving the mechanical characteristics of the base material [1,2].

Among these strategies, the use of polymer composites and other non-metallic materials has been explored as alternative solutions [3]. Furthermore, conventional surface engineering procedures—such as thermal spraying, electroplating, and laser cladding—have been widely applied to prepare metallic coatings and enhance the durability of components [4,5]. However, the high heat input or chemical intensity in these conventional procedures can quickly result in flaws such as phase changes, coating oxidation, and coarse grain size in the heat-affected zone [6]. To overcome these limitations, low-pressure cold spraying (LPCS) has emerged as a promising solid-state deposition method. In order to accomplish bonding, LPCS depends on the severe plastic deformation brought on by supersonic powder particles striking the substrate. This approach offers clear process advantages in the preparation of metal protective coatings since the deposition temperature is significantly below the melting point of the material, avoiding phase transitions caused by high temperatures and residual thermal stress problems [7,8,9].

The synergistic deposition behavior and electrochemical mechanisms of hard and soft particles have made multi-phase composite systems a current research focus in the creation of cold-sprayed anti-corrosion coatings. In their investigation of Fe/Al coatings, Wang et al. [10] discovered that during high-speed deposition, hard Fe particles produce an in situ hammering effect that forces the soft Al phase to experience intensive plastic flow in order to fill the interfacial gaps, greatly increasing the coating density. By creating Zn-Al composite coatings, Zhao et al. [11] showed that the multiphase structure not only lowers porosity but also prevents chloride ion penetration through its corrosion products. By preventing microcrack initiation, Chupong et al. [12] discovered that adding hard TiN particles as a second phase to a soft matrix improves the coating’s overall load-bearing and wear resistance. The fundamental mechanism for attaining long-term cathodic protection of steel substrates is the combination of a dense physical barrier and the sacrificial anode effect of the active phase, according to research by Lu et al. [13]. Therefore, the overall protective performance of coatings can be successfully improved by utilizing the microgalvanic effect and the variations in mechanical characteristics of multiphase particles [14].

The novelty of this study lies in the systematic elucidation of the non-monotonic correlation between Al content and the densification behavior of Fe-Al composite coatings fabricated by low-pressure cold spray (LPCS). While previous literature has primarily focused on the general properties of Fe-Al systems, the underlying competition between the plastic flow of the soft Al phase and the kinetic compaction effect of hard Fe particles remains inadequately addressed. This work distinguishes itself by identifying a critical composition threshold (6 wt.% Al) for optimal structural density and, more importantly, by characterizing a ‘mechanical buffering effect’ at higher Al concentrations (8 wt.%). This buffering effect, which triggers a secondary rise in porosity, provides a new mechanistic explanation for the composition-performance relationship in cold-sprayed metallic composites. By correlating this internal structural evolution with electrochemical protection mechanisms, this research offers a refined theoretical framework for the design of high-performance anti-corrosive coatings on carbon steel [15].

In order to minimize the macroscopic potential difference at the interface and stop rapid corrosion brought on by the large-cathode-small-anode phenomenon caused by dissimilar metal contact, this study chose Fe, which is the same origin as 45 steel, as the coating matrix. In order to serve as a sacrificial anode that dissolves preferentially when the medium penetrates and transfers electrons to the substrate and the Fe matrix, a more negative Al phase was added concurrently. The deposition properties of high-proportion components [16,17,18] or the processes of element diffusion and in situ formation of intermetallic compounds during high-temperature heat treatment [19,20] have been the main topics of previous study on cold-sprayed Fe–Al composite systems. The deposition behavior of systems in their as-deposited condition, without heat treatment, under the control of trace amounts of soft phases, however, has not received much attention. An excessively high Al concentration creates a mechanical buffering effect that absorbs the kinetic energy of particle impacts and lessens the compaction effect of hard particles in low-pressure solid-state deposition, whereas an excessively low Al content is unable to seal pores effectively. Therefore, a critical compositional window must exist where the plastic flow of Al optimally fills the pores without triggering a dominant buffering effect. To accurately capture this dynamic balance, Al contents of 2, 4, 6, and 8 wt.% were selected. This specific narrow gradient was designed to continuously track the transition from under-filling (at extremely low Al levels) to optimal densification, and eventually to the onset of the mechanical buffering effect (at higher Al levels). As a result, this study created Fe-Al composite coatings on the surface of 45 steel with these varying Al mass fractions. The study identifies the ideal composition window for this system, clarifies the synergistic process between physical sealing and sacrificial anode protection, and methodically examines the impact of trace Al gradient fluctuations on the overall performance of the coatings.

## 2. Materials and Methods

### 2.1. Matrix and Powder Materials

The matrix material for this experiment was 45 steel, whose precise chemical composition is presented in Table 1. The 45 steel substrate contains a trace amount of acid-soluble aluminum (Als, 0.023 wt.%). In the industrial steelmaking process, aluminum is typically added as a strong deoxidizer to produce killed steel and to refine the austenitic grain size. Therefore, this trace Als is a normal residual element from the deoxidation process.

**Figure 1:**
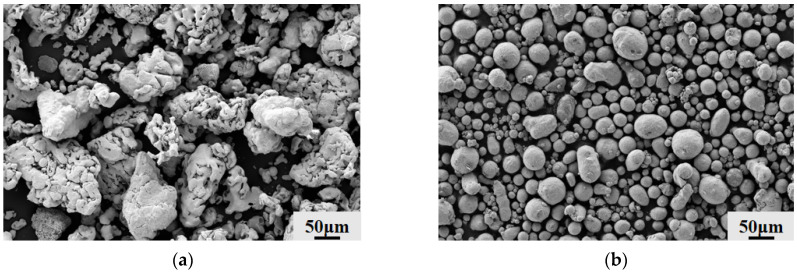

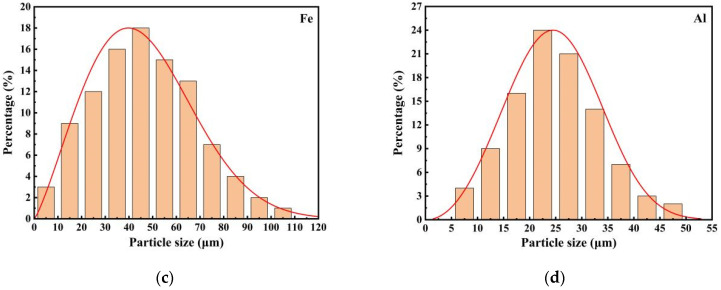
Microscopic morphology and particle size distribution of Fe and Al powders: (**a**) SEM image of Fe powder; (**b**) SEM image of Al powder; (**c**) particle size distribution of Fe powder; (**d**) particle size distribution of Al powder.

The SEM morphological features of the powder materials utilized in the experiment are shown in Figure 1. The analytical grade reduced Fe powder (supplied by Tianjinshi Baishi Chemical Co., Ltd., Tianjing, China) has an uneven overall form, a rough surface, and an inside sponge-like porous structure, as seen in Figure 1a. The Fe powder’s particle size distribution is displayed in Figure 1c, where the average particle size is 46.1 μm. The commercial spherical Al powder (supplied by Beijing Xing Rong Yuan Technology Co., Ltd., Tianjin, China) made by the gas atomization technique has a regular, almost spherical form and a comparatively smooth, dense surface, as seen in Figure 1b. The Al powder’s particle size distribution is displayed in Figure 1d, with an average particle size of 27.7 μm.

### 2.2. Preparation of Fe-Al Composite Coatings

The composite coating in this experiment was prepared using a GDU-3-15 low-pressure cold spray system (Belarusian State University, Minsk, Belarus), which was created and produced by Belarusian State University. Table 2 displays the particular spraying process parameters, including the powder feed rate which was maintained at 15 g/min. The working gas temperature was set within an operational window of 500–600 °C. This range accommodates the dynamic thermal cycling of the equipment while ensuring that the hard Fe particles reach their critical velocity and the soft Al particles achieve sufficient plasticization without melting. Similarly, the spraying distance was maintained within a narrow 10–15 mm window. This specific stand-off range is optimal for low-pressure cold spray to maximize particle impact kinetic energy while minimizing the adverse deceleration effects caused by the bow-shock wave near the substrate surface. The 45 steel substrate’s surface was prepared by sandblasting using brown fused alumina as the abrasive before spraying to guarantee a strong adhesion between the coating and the substrate. In order to provide enough mechanical interlocking sites for coating deposition, this technique raised surface roughness and eliminated native oxide scale from the substrate surface.

In order to guarantee uniform distribution, reduced Fe powder and spherical Al powder were combined in mass ratios of 98:2, 96:4, 94:6, and 92:8, respectively (the corresponding composite coatings were denoted as 2Al, 4Al, 6Al, and 8Al). The mixture was mechanically mixed for two hours. Fe-Al composite coatings with different Al ratios were then deposited onto the pretreated substrate surface by progressively loading the produced powder combinations into the powder feeder.

### 2.3. Microstructural Characterization and Mechanical Property Testing

Electric discharge wire cutting was used to cut four sets of coated specimens into blocks measuring 10 mm by 10 mm. After being gradually sanded with SiC sandpaper (80–3000 grit), they were polished with a metallographic polishing machine, ultrasonically cleaned in anhydrous ethanol, and allowed to air dry.

A white-light interferometer (WLI, SuperView W3, Chotest Technology Inc., Shenzhen, China) was used to measure the coating’s three-dimensional surface topography and quantitatively evaluate the surface roughness before polishing. Specifically, to determine the arithmetical mean height (Ra), a standard Gaussian filter was applied to separate the high-frequency roughness profile from the low-frequency waviness. Based on the optical field of view, the evaluation length for the extracted 2D profiles was set to 979.0 μm. To ensure statistical reliability, five independent measurements were performed at randomly selected locations on each sample surface, and the average Ra values along with their standard deviations were recorded. Furthermore, an optical microscope (OM) and a scanning electron microscope (SEM) were used to observe the surface and cross-sectional microstructures of the coatings. An energy-dispersive X-ray spectrometer (EDS) was utilized to analyze the elemental composition, and Image-Pro Plus 6.0 software was employed to analyze and calculate the thickness and porosity variations across the four coating groups. The phase structure of the coatings was examined using an X-ray diffractometer (XRD, D8 ADVANCE, Bruker Corporation, Karlsruhe, Germany) with a Cu-target Kα radiation source (λ = 0.15406 nm). The test was conducted in a continuous scanning mode with a 2θ range of 20–100°, a scanning speed of 4°/min, and a step size of 0.02°. The obtained XRD diffraction patterns were subjected to background subtraction and smoothing before being analyzed using MDI Jade 6 software for phase identification and peak indexing. The microhardness of the coated surface was measured using an HV-0.2 micro-Vickers hardness tester with a load of 1.96 N (0.2 kgf), a holding period of 15 s, and a spacing of 1 mm between test points. For every set of coated samples, eight parallel measurements were made, and the average value was computed after outliers were eliminated. Origin 2024 software was used to statistically analyze and plot the experimental data.

### 2.4. Electrochemical Corrosion Performance Testing

First, copper wires were soldered to the back of the ground and polished block-shaped coated specimens. Subsequently, the specimens were cold-embedded using a commercial acrylic cold mounting resin (Model XBH-30, Shanghai Xinbiao Testing Machine Co., Ltd., Shanghai, China). The non-working surfaces and the sample edges were completely sealed by the insulating resin to prevent any solution penetration or galvanic corrosion, leaving only a precisely measured 10 mm × 10 mm working area of the coating exposed to the test media. For every set of parameters, three parallel specimens were constructed to guarantee the repeatability of the experimental data.

A typical three-electrode system was used for chemical testing on a CHI660D electrochemical workstation: the coated specimen was used as the working electrode (WE), a platinum sheet as the counter electrode (CE), and a saturated calomel electrode (SCE) as the reference electrode (RE). Testing was done in an ambient setting at room temperature using a naturally aerated 3.5 wt.% NaCl solution as the corrosion medium. The pH of the solution was not artificially controlled, maintaining a natural, unbuffered pH of approximately 7.0. After immersing the sample in the cell for 1800 s to achieve a stable open-circuit potential (OCP), electrochemical impedance spectroscopy (EIS) testing was carried out using a sinusoidal AC excitation signal with an amplitude of 5 mV within the frequency range of 10^−2^ to 10^5^ Hz; dynamic potential polarization curves were measured at a scan rate of 1 mV/s and within the voltage range of −2 to 0 V. This relatively wide potential range was specifically selected to fully capture the comprehensive cathodic reduction processes (including both oxygen reduction and hydrogen evolution) and the complete anodic active dissolution behaviors of the multiphase system (including the preferential dissolution of the active Al phase and the subsequent behavior of the Fe matrix).

## 3. Results and Discussion

### 3.1. Analysis of the Original Surface Topography and Roughness of the Coating

**Figure 2:**
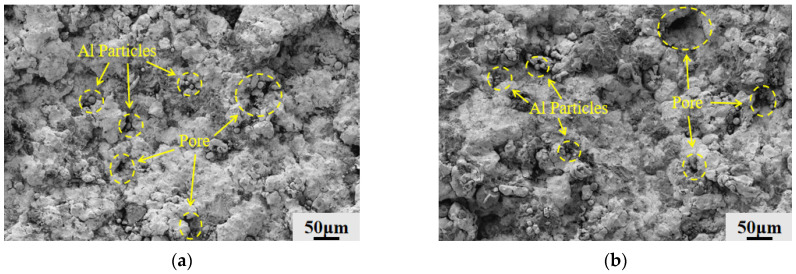

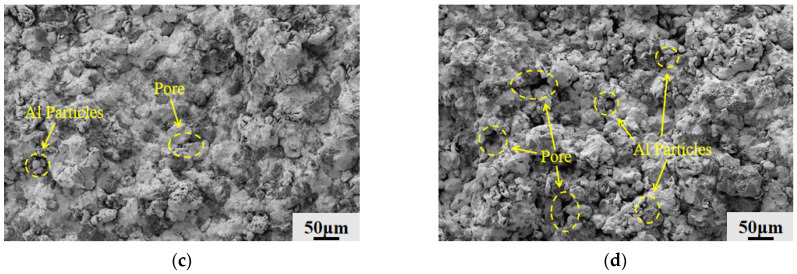
Microscopic morphology of the coated surface (before polishing): (**a**) 2Al; (**b**) 4Al; (**c**) 6Al; (**d**) 8Al. (the yellow dashed circles and arrows in the figure indicate undeformed spherical Al particles and pore defects).

The surface topography of Fe-Al composite coatings with varying Al concentrations as they were applied before grinding is seen in Figure 2. The degree of plastic deformation following particle impact is the main factor influencing the coating’s densification during the cold spray solid-state deposition process [21]. The surfaces of the 2Al and 4Al coatings are comparatively rough, displaying typical brittle deposition features, as seen in Figure 2a,b. There are many Al particles on the surface that have not experienced significant deformation and still have their original spherical shape because the soft phase’s low Al concentration limits the particles’ overall degree of plastic deformation. A high density of pores on the coated surface results from these undisturbed particles’ inability to spread out and fill the gaps. The coating is vulnerable to microcrack initiation at stress concentration areas due to the weak mechanical connection between particles [22,23]. On the other hand, as Figure 2c illustrates, the 6Al coating’s surface pores have mostly vanished, leaving only a few isolated micro-pores, and the deposited surface is smooth. The Al particles at this composition experience enough plastic deformation and spreading upon collision, nearly completely losing their initial spherical shape. In order to achieve tight mechanical interlocking and prevent the formation of voids and cracks, the fully deformed soft particles successfully fill the voids and fuse well with the Fe. But as Figure 2d illustrates, flaws on the 8Al coating’s surface resurface, with a large number of gaps that only contain a few Al particles. This phenomenon is mainly caused by the system’s abnormally large fraction of the soft phase (Al), which lowers the relative proportion of the hard phase (Fe). Subsequent high-velocity hard Fe particles are essential for compacting the underlying deposited layer during the continuous cold spray deposition procedure [24]. The kinetic energy of successive hits cannot be efficiently transformed into the deformation energy needed for coating compaction when the soft Al layer is too thick, creating a substantial buffering effect. This leads to a loose accumulation and weak interparticle bonding, which re-induces a pore network and possible cracks at the deposition interface [25]. As a result, 6Al is the ideal composition window for controlling powder plastic deformation and bonding behavior, guaranteeing both adequate hard matrix maintenance to ensure effective compaction during deposition and sufficient soft particle deformation to fill voids.

**Figure 3:**
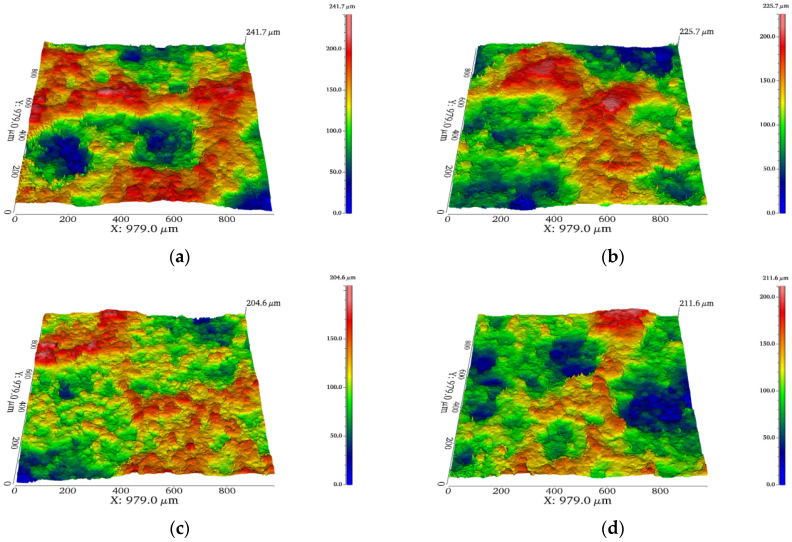
Three-dimensional topography of the coated surface (before polishing): (**a**) 2Al; (**b**) 4Al; (**c**) 6Al; (**d**) 8Al.

**Figure 4:**
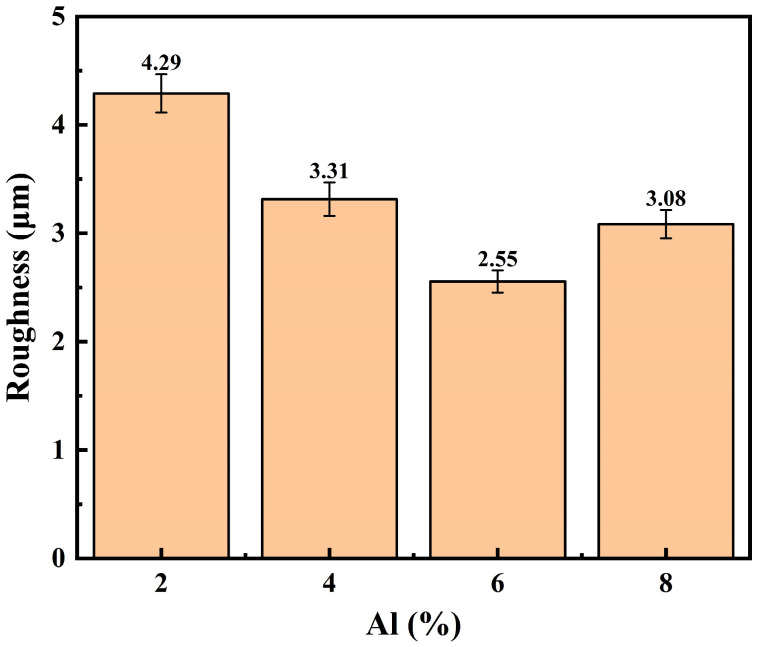
Average surface roughness of the coating (before grinding).

Figure 3 and Figure 4 depict the three-dimensional topography of the coatings in each group prior to polishing, together with the related surface roughness test results, in order to further quantify the particle accumulation and topographical differences on the coating surfaces. The three-dimensional profiles of the 2Al and 4Al coatings display several noticeable peaks and deep troughs with notable height fluctuations, as seen in Figure 3a,b. According to the findings in Figure 4, the 4Al coating’s roughness drops to 3.31 μm, whereas the 2Al coating has the highest roughness at 4.29 μm. The low content of soft phases prevents the particles from undergoing enough plastic flow to fill the gaps when impacting the substrate, and the original contours of many undeformed particles are retained on the surface, resulting in a relatively high overall roughness. This is consistent with the brittle deposition characteristics seen in the microstructure. The distribution of peaks and valleys on the surface of the 6Al coating in Figure 3c dramatically decreased when the Al concentration reached 6 wt.%, and the overall three-dimensional shape smoothed out. Concurrently, 6Al’s roughness in Figure 4 dropped to 2.55 μm, the lowest of the four groups. The ideal coating density and homogeneity were achieved by the soft Al particles on the surface undergoing adequate plastic deformation and spreading, which successfully filled the spaces between the hard matrix and smoothed the surface profile. However, Figure 3d demonstrates that the 8Al coating’s three-dimensional morphology once more displays deep localized pits and the degree of surface undulation has increased; Figure 4’s results also show that the coating’s roughness has recovered, increasing to 3.08 μm. This suggests that the hard Fe particles’ subsequent compaction effect is weakened by an excess of soft Al phase in the system. The kinetic energy from the impact of the sprayed particles cannot be efficiently transformed into the deformation energy needed to compact the underlying coating because of the buffering effect created by the thick soft layer. This leads to loose packing between particles and the reformation of a local pore network, which ultimately increases macroscopic surface roughness. 6Al is the perfect composition window for balancing plastic deformation and particle compaction, producing the best surface quality, according to a thorough investigation of the trends in 3D morphology and roughness.

### 3.2. Microstructural Evolution and Phase Composition of the Coating

#### 3.2.1. Cross-Sectional Morphology and Densification Behavior of the Coating

**Figure 5:**
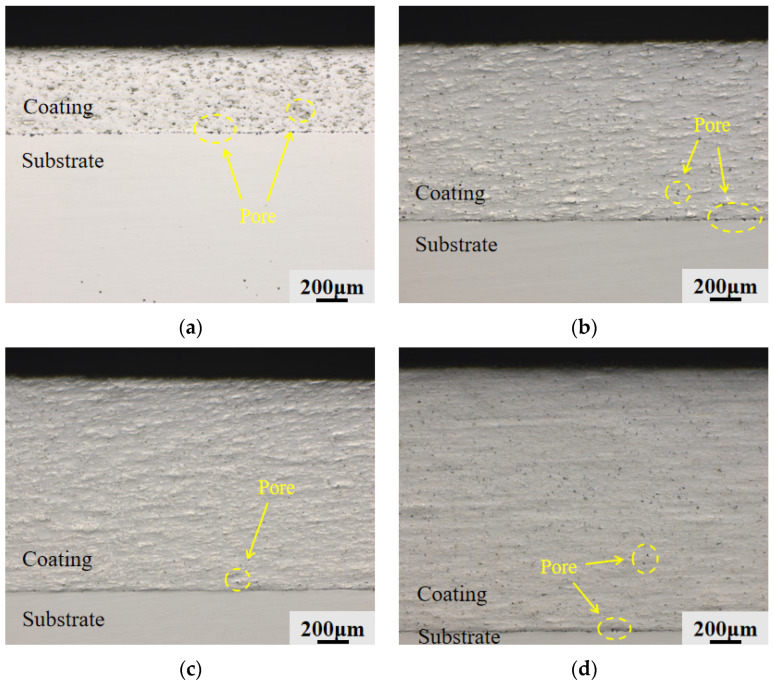
Microstructural morphology of the coating cross-sections: (**a**) 2Al; (**b**) 4Al; (**c**) 6Al; (**d**) 8Al.

The microstructural cross-sectional pictures of the four groups of Fe-Al composite coatings following polishing and grinding are displayed in Figure 5. The mechanical binding interface that forms between the cold-sprayed deposit on top of the underlying 45 steel substrate is clearly depicted in the image. All four groups of coatings attained continuous coverage on the substrate, as seen in the figure, and the apparent thickness of the coatings showed a distinct upward trend as the system’s Al concentration increased. The 2Al and 4Al coatings in Figure 5a,b have many distinct black porous regions; some of these pores are near the bonding interface, suggesting that the particles did not achieve fully compact stacking during the deposition process, according to an examination of the microstructural features within the coatings and at the interfaces. The coating cross-section became the densest with a notable decrease in macroscopic black voids when the Al content reached 6 wt.%, as seen in Figure 5c. Additionally, there were no cracks or delamination at the coating-substrate contact, indicating strong interlayer adhesion. On the other hand, the internal structure of the coating became loose once more and dispersed voids reappeared in the 8Al coating cross-section, as seen in Figure 5d.

**Figure 6:**
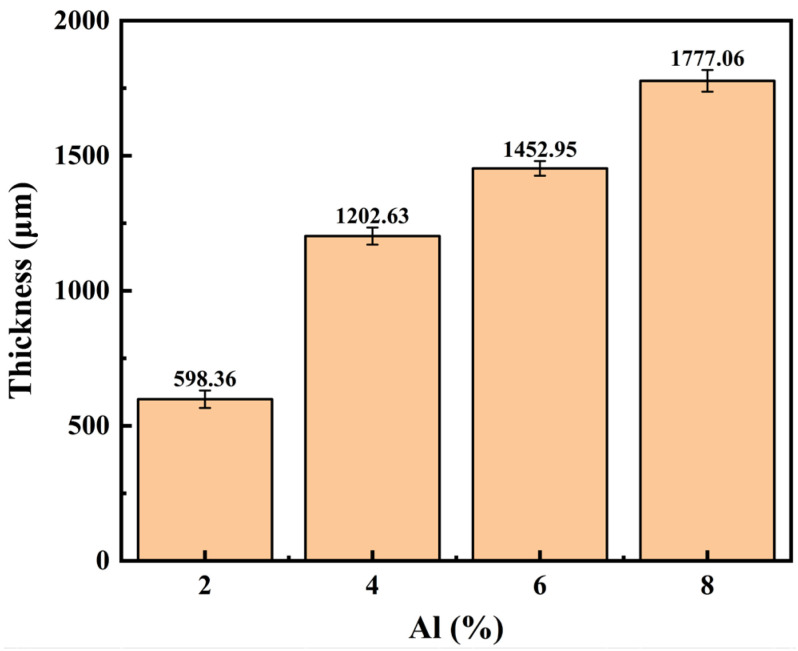
Average cross-sectional thickness of the composite coatings in each group.

The cross-sectional thickness variations of each group’s coatings are displayed in Figure 6. According to the findings, the coating thickness increases steadily as the system’s Al content rises. With a thickness of 598.36 μm, the 2Al coating was the thinnest. The ability of the particles to undergo plastic deformation has a significant impact on the deposition efficiency of powders in low-pressure cold spraying [26]. Hard Fe particles find it difficult to produce enough deformation upon collision with the substrate to establish mechanical interlocking because there are few soft phases in the 2Al powder system. A low overall deposition efficiency is the result of many particles rebounding and detaching. The thickness of the 4Al coating rises to 1202.63 μm as the Al content rises because there are more soft phases, which encourage particle deformation and adhesion. The coating thickness was 1452.95 μm at the 6Al composition. At this stage, a large number of soft Al particles experienced plastic spreading upon high-speed impact, acting as an efficient bonding phase between the hard skeletal particles. In addition to removing pores, this spreading greatly decreased the rebound loss of later powders, preserving a dense and effective deposition process. The cross-sectional thickness of the 8Al coating attained its highest value among the four groups at 1777.06 μm when the Al content reached 8 wt.%. The cross-sectional morphology in Figure 5 illustrates that the abrupt increase in the thickness of the 8Al coating is directly linked to the evolution of its internal structure toward a more porous state rather than merely reflecting a proportionate increase in the amount of deposited material. On the deposition surface, an excess of soft Al phase created a thick buffer layer that reduced the compaction effect of the underlying layer by absorbing the kinetic energy of succeeding hard Fe particles. The coating expanded in macroscopic volume as a result of this loose particle collection, giving the cross-section an unusually high measured thickness.

**Figure 7:**
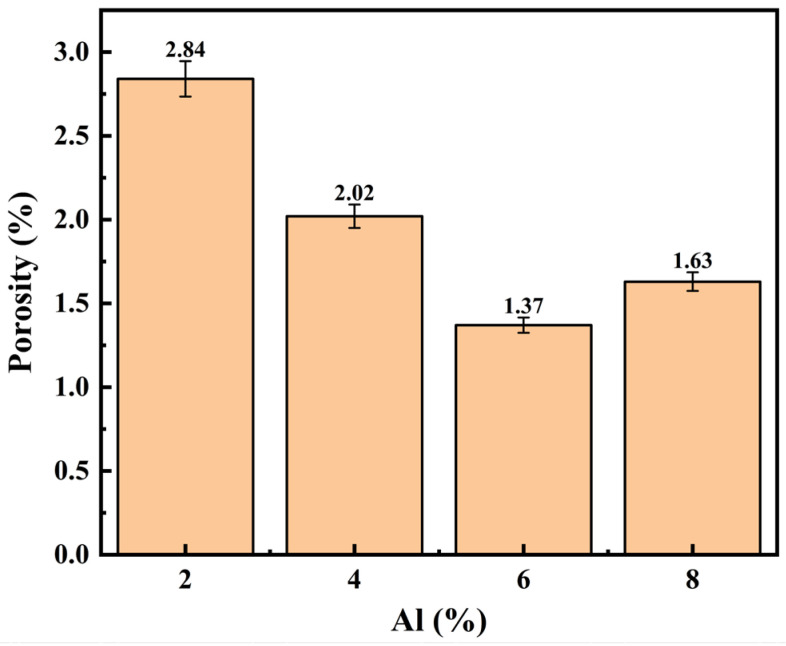
Average porosity of the composite coating surfaces in each group.

The statistical findings for each group’s coating surface porosity are displayed in Figure 7. At 2.84%, the porosity of the 2Al coating was the highest. There were many unclosed mechanical spaces between the hard Fe particles as a result of the powder system’s abnormally low soft phase fraction, which prevented the particles from undergoing enough plastic flow under low-pressure impact. The porosity of the 4Al coating dropped to 2.02% when the soft Al phase was added, improving the particles’ capacity to deform in coordination. The coating’s internal porosity decreased to at least 1.37% when the Al content hit 6 wt.%. This shows that an appropriate proportion of hard Fe particles maintains strong impact kinetic energy in the substrate, fully compacting the spread Al phase and optimizing the internal density of the coating, while an appropriate amount of Al particles undergoes sufficient ductile deformation and spreading during high-speed impact, effectively filling the microscopic voids within the hard matrix as a soft bonding phase. The porosity increased to 1.63% when the Al concentration was raised to 8 wt.%. This suggests that an excess of the Al phase created a comparatively thick soft area inside the coating, which had a major buffering impact on particles that were deposited later. The hard Fe particles’ impact compaction effect was directly decreased by this buffering, which left the underlying particles with insufficient compressive force. As a result, the internal packing state changed from dense to loose, which led to an additional rise in porosity flaws.

The densification of Fe-Al coatings depends on the interaction between the plastic flow of the soft Al phase and the compaction from hard Fe particles. At low Al concentrations (2–4 wt.%), the ductile phase is insufficient to fill the gaps in the Fe skeleton. At 6 wt.% Al, the Al particles plastically deform upon high-speed impact and act as a binder to seal the pores. However, when the Al content reaches 8 wt.%, the excess soft phase creates a thick buffer layer. This layer absorbs the kinetic energy of the incoming Fe particles and weakens their compaction effect on the underlying coating, causing the porosity to increase again.

#### 3.2.2. Microstructure and Elemental Distribution of the Coating

**Figure 8:**
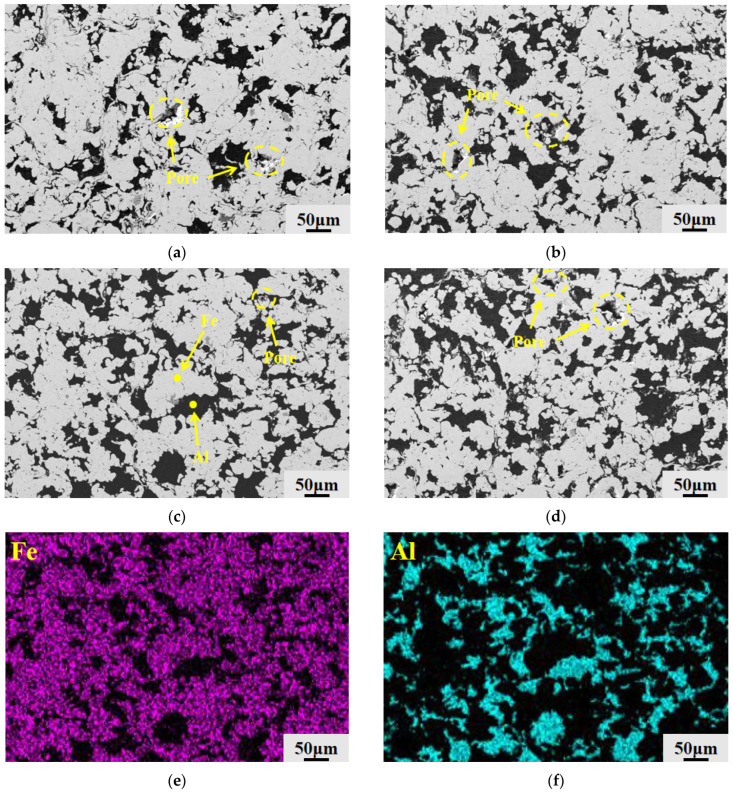
SEM micrographs of the composite coating surface and EDS elemental distributions of the 6Al coating: (**a**) 2Al; (**b**) 4Al; (**c**) 6Al; (**d**) 8Al; (**e**) Fe elemental distribution of the 6Al coating; (**f**) Al elemental distribution of the 6Al coating (the yellow dashed circles and arrows in the figure indicate pore defects and regions with different phases).

Figure 8 displays the EDS surface scan results at 6 wt.% Al composition as well as SEM micrographs of the surfaces of the Fe-Al composite coatings from each group following grinding and polishing. The microstructural composition of the coating can be visually identified by using the contrast differences in the material under the electron microscope. The lighter-colored regions in the figure represent the hard Fe phase, the dark gray regions represent the soft Al phase that has undergone plastic deformation, and the deep black micro-regions represent micro-pores within the coating as well as delamination pits caused by mechanical tearing due to poor grain bonding during the metallographic grinding and polishing process.

The surfaces of the 2Al and 4Al coatings had many huge, deep black defect patches, as seen in Figure 8a,b, according to a comparison of the morphologies of the four coating groups. Following the densification behavior established in Section 3.2.1, the lack of ductile Al in these coatings prevents complete pore filling, leaving poorly bonded particles that easily detach during polishing. As shown in Figure 8c, when the Al content increases to 6 wt.%, the dark black areas on the coated surface are greatly diminished, and the structure becomes highly dense. The dark gray Al phase uniformly fills the interfaces of the light-colored Fe matrix, interweaving like a network. However, as depicted in Figure 8d, a significant number of free-standing dark black flaws reappear on the surface of the 8Al coating, even though the distribution area of the Al phase has further expanded. This reappearance of spalling defects confirms the mechanical buffering effect of the excess Al, which weakens interparticle bonding and causes a secondary rise in porosity.

As shown in Figure 8e,f, the EDS surface scans of the dense 6Al coating further confirm its phase distribution and elemental composition. Correlating these maps with the SEM morphology shows that Al concentrates primarily in the plastically deformed dark gray networks, while the light-colored matrix consists entirely of Fe. The soft Al phase distributes evenly throughout the hard Fe framework without any macroscopic segregation or large-scale aggregation. These elemental maps directly demonstrate the tight spatial interlacing and mechanical interlocking of the two phases achieved during cold spray deposition.

**Figure 9:**
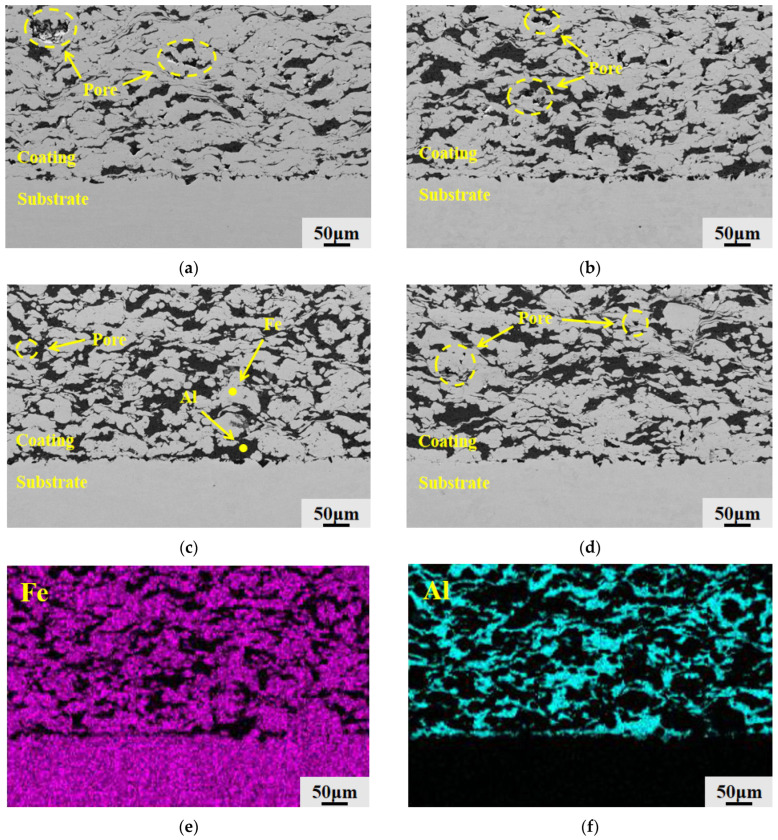
SEM micrographs of the cross-section of the composite coating and EDS elemental distributions of the 6Al coating: (**a**) 2Al; (**b**) 4Al; (**c**) 6Al; (**d**) 8Al; (**e**) Fe elemental distribution in the cross-section of the 6Al coating; (**f**) Al elemental distribution in the cross-section of the 6Al coating (the yellow dashed circles and arrows in the figure indicate pore defects and regions with different phases).

To explore the microstructural evolution in the depth direction, Figure 9 presents the cross-sectional SEM morphologies of the four coatings and the corresponding EDS maps for the 6Al composition. The cross-sections clearly reveal the particle deformation induced by the unidirectional impact of cold spraying. As shown in Figure 9a–d, the dark gray Al phase exhibits a flattened morphology, spreading parallel to the interface in an interwoven lamellar pattern. Following the densification mechanism described in Section 3.2.1, the inadequate Al content in the 2Al and 4Al coatings results in a discontinuous lamellar network with numerous open spaces between the Fe particles. When the Al content reaches 6 wt.%, these lamellae become dense and continuous, interweaving to eliminate the voids in the Fe matrix. However, a thick area of aggregated Al appears in the cross-section of the 8Al coating. This excess Al triggers the aforementioned mechanical buffering effect, causing interlaminar gaps and loose holes to reemerge.

The EDS maps in Figure 9e,f further confirm the composition of this interwoven structure. In the cross-section of the dense 6Al coating, the flattened dark network consists primarily of Al, while the surrounding light-colored matrix is composed of Fe. The two phases form a uniform, interlocked structure without macroscopic segregation, confirming the effective pore sealing and mechanical interlocking achieved at this optimal concentration.

**Figure 10:**
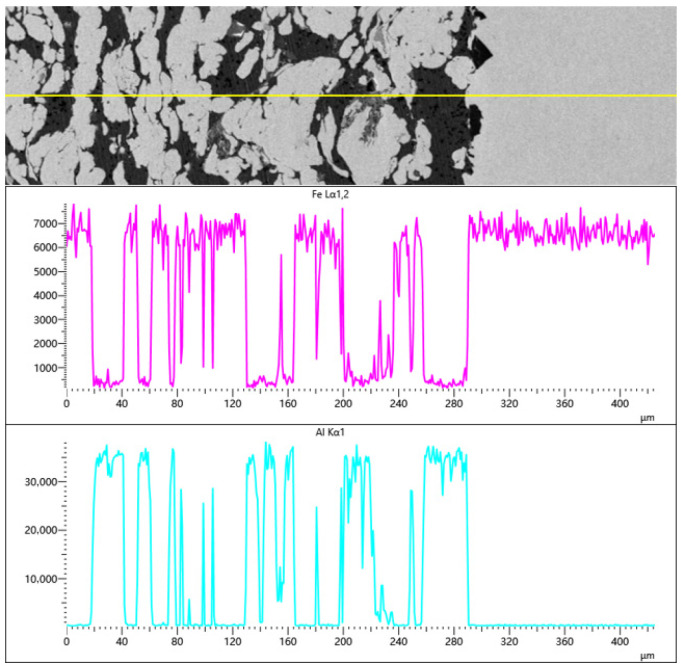
EDS line scan results across the cross-section and interface of the 6Al composite coating (the solid yellow line indicates the scan path).

As shown in Figure 10, the EDS line-scan spectra taken vertically across the interface of the 6Al coating reveal the bonding process with the 45 steel substrate. These elemental transition characteristics help determine the bonding mechanism in cold spray deposition. The elemental concentrations show a sharp, step-like change across the boundary. Because the substrate is 45 steel, the Fe signal maintains a steady high level upon entering the substrate, while the Al signal rapidly drops to zero. This sharp concentration gradient and narrow transition zone reflect the solid-state nature of low-pressure cold spray. Since the carrier gas temperature is well below the melting point of the powders, the particles lack the activation energy required for long-range atomic diffusion during the brief impact period. As a result, no significant macroscopic elemental diffusion or large-scale metallurgical reactions are observed between the coating and the substrate under the current resolution. Therefore, the coating’s adhesion is primarily dependent on local plastic deformation and mechanical interlocking brought on by the particles’ rapid impact on the surface, which is generally considered the dominant bonding mechanism in low-pressure cold spray without subsequent heat treatment [27,28].

Within the coating, the Fe and Al signal intensities show high-frequency, alternating oscillations, where the peaks of the Fe signal correspond to the troughs of the Al signal. These strong signal variations result from the mechanical mixing and the interlaced lamellar structure formed during the layer-by-layer deposition of the composite powder. The line-scan data indicate that no significant alloying occurs during deposition, and the Fe and Al particles retain their original chemical identities at the elemental scale.

#### 3.2.3. Phase Composition of the Coating

**Figure 11:**
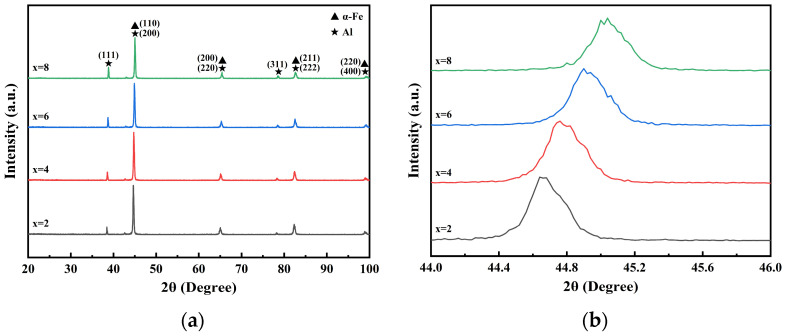
XRD patterns of the Fe-xAl composite coating surface: (**a**) overall diffraction pattern; (**b**) magnified view of the 44–46° region.

The XRD patterns of the surfaces of four groups of Fe-Al composite coatings with varying Al concentrations are displayed in Figure 11. Only the body-centered cubic α-Fe phase and the face-centered cubic Al phase were found in the four groups of coatings, according to an analysis of Figure 11a. The (110), (200), (211), and (220) crystal planes are the primary diffraction peaks of α-Fe, whereas the (111), (200), (220), (311), (222), and (400) crystal planes are the distinctive diffraction peaks of Al. Within the detection limits of the XRD equipment, no significant diffraction peaks corresponding to metal oxides or Fe–Al intermetallic compounds were identified over the scanning range. This indicates that the low-pressure cold spray method exhibits typical solid-state deposition characteristics, where no obvious phase transformation or large-scale thermal activation reactions occurred. However, the presence of trace amounts of minor phases below the detection threshold (typically < 2–5%) cannot be entirely ruled out. As a result, the coating completely preserves the powder’s initial phase composition. The coating’s two phases continue to be only mechanically combined. The fundamental material basis for the active Al phase to function as a sacrificial anode in later corrosive settings is provided by this total preservation of the original phase composition. According to the experimental formulation, the relative intensity of the Al phase diffraction peaks regularly increases as the system’s Al content rises, whereas the relative intensity of the α-Fe phase diffraction peaks declines.

Additionally, as Figure 11b illustrates, the distinctive diffraction peaks of both Al and α-Fe shift somewhat toward higher angles with increasing Al content. Bragg’s law states that an increase in the diffraction angle θ results in a decrease in the interplanar distance d (where d is the interplanar spacing, θ is the diffraction angle, and λ is the X-ray wavelength). Subsequent high-speed hits of hard Fe particles during continuous cold spray deposition have a severe mechanical shot peening effect on the underlying coating, causing the unit cells to compress perpendicular to the surface and creating a large residual compressive stress [29]. The system’s plastic flow-capable regions grow as the percentage of soft Al components rises; the impact of hard Fe particles produces stronger localized compression, which transforms kinetic energy into plastic strain energy, increasing internal dislocation density and causing deeper lattice distortion. The buildup of high Al content and crystal defects, along with this residual compressive stress, eventually show up macroscopically as a general rightward shift of distinctive diffraction peaks.

### 3.3. Microhardness Analysis

**Figure 12:**
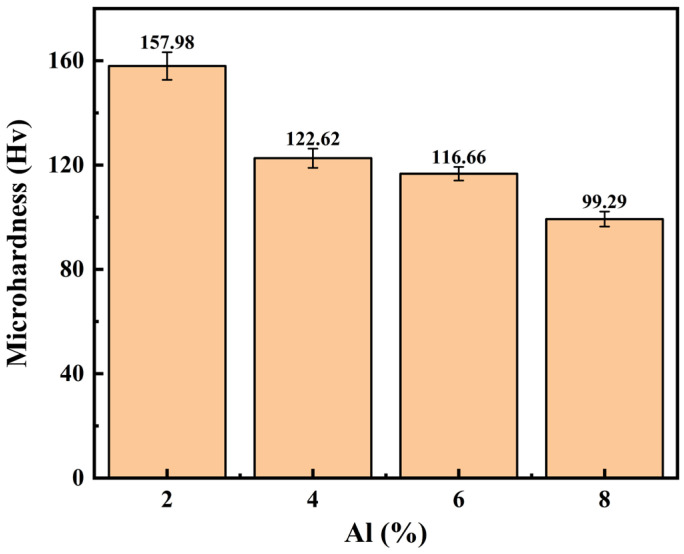
Average micro-Vickers hardness of the Fe-Al composite coatings in each group.

As shown in Figure 12, the average micro-Vickers hardness of the coatings decreases monotonically as the Al content increases. The 2Al coating exhibits the maximum hardness at 157.98 HV, while the hardness of the 8Al coating drops to 99.29 HV.

The hardness of the Fe-Al composite coatings (157.98–99.29 HV) is consistently lower than that of cold-sprayed pure iron coatings, which typically exceed 200 HV due to intense work hardening during impact [30]. Conversely, it remains significantly higher than the microhardness of cold-sprayed pure aluminum coatings (approximately 30–60 HV) [31]. This intermediate hardness confirms that the Fe matrix maintains its overall structural integrity, while the soft Al phase effectively fills the inter-particle gaps.

The macroscopic hardness of the composite coatings ultimately depends on the intrinsic hardness of the phases, coating density, and strain hardening. According to the rule of mixtures, increasing the proportion of soft Al directly raises the volume fraction of the low-hardness phase, which explains the monotonic decrease from the 2Al to 8Al coatings and the reduction in overall load-bearing capacity [32]. Furthermore, cold spraying induces severe plastic deformation and work hardening [33]. In the 2Al system, the high proportion of hard Fe particles causes intense mechanical compaction, accumulating high dislocation densities and residual compressive stresses that maximize local hardness. However, as the Al concentration rises, the growing network of soft Al triggers the mechanical buffering effect described in Section 3.2.1. This buffering limits the compaction from subsequent particles, reducing the total work hardening and accelerating the hardness decline. Although the 6Al coating achieves the highest structural density, its hardness remains lower than that of the 2Al and 4Al coatings. This confirms that the intrinsic hardness loss from the additional soft phase completely offsets the hardness gain from improved density. Finally, the 8Al coating exhibits the lowest hardness because the extensive soft phase and the increased porosity severely disrupt the structural continuity.

### 3.4. Electrochemical Corrosion Behavior and Protection Mechanisms

**Figure 13:**
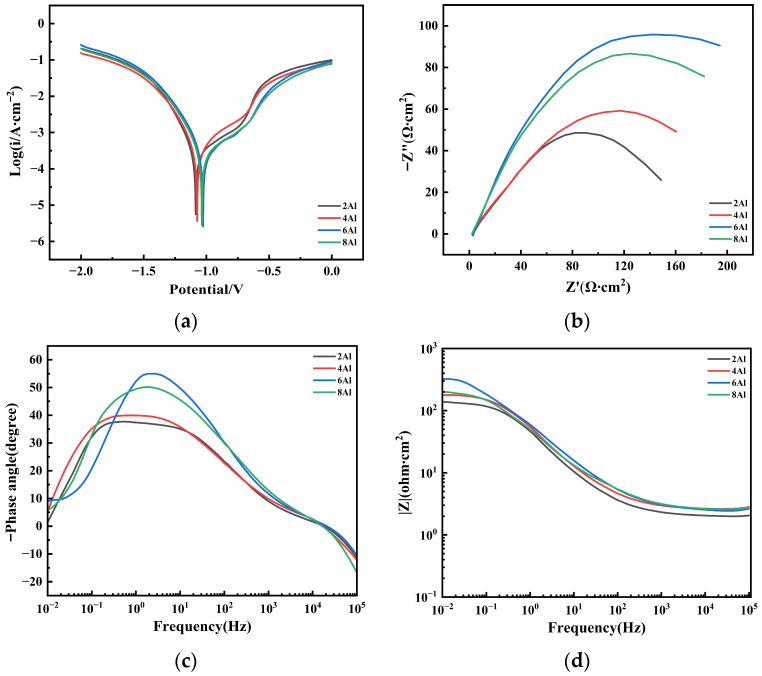
Stripping potential polarization curves and electrochemical impedance spectra of various Fe-Al composite coatings in a 3.5 wt.% NaCl solution: (**a**) polarization curves; (**b**) Nyquist plots; (**c**) Bode phase angle plots; (**d**) Bode modulus plots.

To assess their protective effectiveness, the electrochemical behavior of the Fe-Al composite coatings was investigated in a 3.5 wt.% NaCl solution. As shown in Figure 13a and Table 3, the dynamic polarization curves of all four coating groups exhibit typical active dissolution features without a passivation region, indicating that activation polarization controls the anodic reaction. Compared to the 2Al coating, increasing the Al content initially improves corrosion resistance, as evidenced by a positive shift in corrosion potential and a decrease in corrosion current density, before deteriorating at higher concentrations. The 6Al coating exhibits the optimal corrosion resistance, featuring the lowest corrosion current density of 2.237 × 10^−4^ A/cm^2^, the most positive corrosion potential of −0.69462 V, and the highest overall polarization resistance of 5122.9 Ω·cm^2^. Here, Rp explicitly represents the macroscopic resistance to the overall corrosion process derived from the potentiodynamic polarization curves. Consistent with the microstructural analysis in Section 3.2.1, this optimal Al content effectively seals the pores, blocking electrolyte penetration. According to the mixed potential theory, this physical sealing alters the corrosion process both thermodynamically and kinetically. Thermodynamically, the positive shift of E_corr_ in the 6Al coating indicates a reduced driving force for anodic dissolution. Kinetically, the densified matrix minimizes the electrochemically active surface area and restricts electrolyte diffusion, which significantly increases the activation overpotential for charge transfer. Thus, the superior corrosion resistance of the 6Al coating stems from a combined effect of thermodynamic surface stabilization and strong kinetic hindrance. When the Al content reaches 8 wt.%, the corrosion potential drops to −0.70343 V and the current density rises to 2.314 × 10^−4^ A/cm^2^. This decline occurs because the aforementioned buffering effect increases porosity, creating pathways for the corrosive medium.

Electrochemical impedance spectroscopy further verifies these findings. As shown in the Nyquist plots in Figure 13b, the radius of the capacitive arc first increases and then decreases with rising Al content. A larger arc radius corresponds to higher charge transfer resistance and a lower corrosion rate [34]. The 6Al coating presents the largest capacitive arc, confirming its superior interfacial reaction resistance. Similarly, the Bode plots in Figure 13c,d reveal that the 6Al coating possesses the highest phase angle and maximum impedance modulus, corroborating its optimal protective performance.

**Figure 14:**
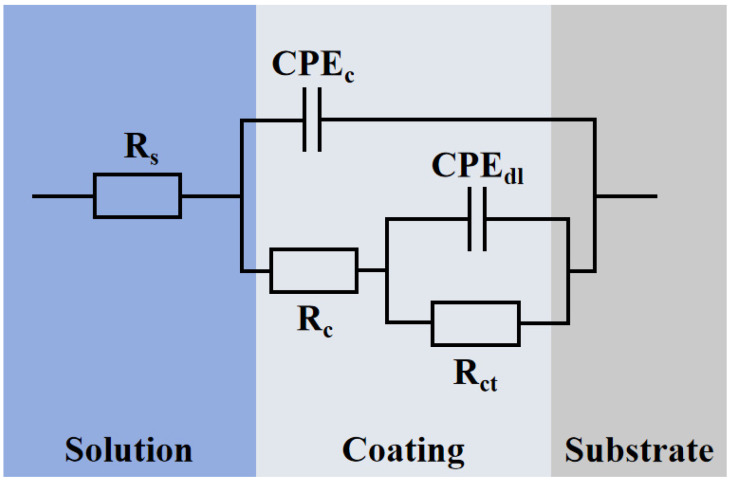
Equivalent circuit model for fitting the EIS data of the Fe-Al composite coating.

To further examine the electrochemical performance, the double-time-constant equivalent circuit model in Figure 14 was used to fit the impedance data. Here, Rs, Rc, and Rct represent the solution resistance, coating pore resistance, and interfacial charge transfer resistance, respectively. A constant phase element (CPE) replaces the ideal capacitance to account for the non-ideal capacitive response caused by surface porosity and phase heterogeneity [35]. As shown in Table 4, the deviation of the exponents nc and ndl from 1 confirms this structural non-uniformity. With increasing Al content, both Rc and Rct initially rise and then decline, peaking at 6 wt.% Al and reaching their lowest at 2 wt.% Al. The maximum Rct indicates the highest resistance to interfacial charge transfer, while the peak Rc confirms that the dense 6Al network effectively blocks electrolyte penetration. Unlike the total macroscopic polarization resistance (R_p_) evaluated earlier, this R_ct_ specifically reflects the local electron transfer kinetics at the electrolyte/matrix interface. Although they measure distinct physical properties, both parameters independently confirm the optimal anti-corrosion performance of the 6Al coating. Conversely, the capacitance values (CPEc and CPEdl) decrease and then increase, reaching a minimum at 6 wt.% Al. Since a lower capacitance indicates a smaller active area in contact with the electrolyte, this result aligns with the polarization curve findings, confirming the superior protection of the 6Al coating.

Based on the microstructural and electrochemical analyses, the corrosion protection of the Fe-Al coatings relies on physical shielding and sacrificial anode effects. During initial immersion, the structural density dictates the corrosion resistance. As established in Section 3.2.1, the optimal Al content minimizes porosity, forming a continuous physical barrier against the electrolyte. If trace electrolyte penetrates the residual pores, the more electronegative Al phase dissolves preferentially. This acts as a sacrificial anode, galvanically protecting the cathodic Fe skeleton and the steel substrate. Ultimately, the 6Al coating provides the best corrosion resistance by balancing physical densification and electrochemical protection, a conclusion that strictly corroborates the microstructural and porosity measurements.

## 4. Conclusions

Low-pressure cold spray technology was used to create Fe-Al composite coatings with different aluminum contents on the surface of 45 steel. The microstructure, mechanical characteristics, and corrosion resistance of the composite coatings were thoroughly examined in relation to the aluminum content (2, 4, 6, and 8 wt.%). The primary conclusions reached were as follows:

(1) The 45 steel substrate and the low-pressure cold-sprayed Fe-Al composite coating show typical mechanical bonding. No significant phase transformation or oxidation was detected by XRD during the deposition process, and the coating largely preserves the initial α-Fe and Al phases of the raw powders. The porosity and surface roughness of the coating show a trend of first reducing and then increasing as the system’s Al concentration rises. The lowest surface roughness (2.55 μm) and best porosity (1.37%) are achieved when the Al content is 6 wt.% because the soft Al particles experience enough plastic flow to efficiently fill the spaces between the rigid matrix. A mechanical buffering effect is triggered by excessive Al addition (8 wt.%), which weakens the continuous compaction during particle deposition and causes porosity defects to reappear.
(2) With an increase in Al content, the microhardness of Fe-Al composite coatings shows a monotonically declining pattern, falling from 157.98 HV for the 2Al coating to 99.29 HV for the 8Al coating. The higher volume fraction of the low-hardness Al phase is the main cause of this. Simultaneously, the kinetic energy from particle hits is absorbed by the interconnected soft network, which lessens the hard Fe particles’ compaction effect on the substrate and lowers the system’s overall work hardening.
(3) The corrosion resistance of the composite coatings first rose and then fell with increasing Al concentration in a 3.5 wt.% NaCl solution. With the highest pore resistance and charge transfer resistance, the most positive corrosion potential (−0.69462 V), and the lowest corrosion current density (2.237 × 10^−4^ A/cm^2^), the 6Al composite coating demonstrated the best resistance to electrochemical corrosion. Physical shielding and micro-galvanic sacrificial anode protection work in concert to prevent corrosion. The exceptionally high density of the 6Al coating maximally blocks the longitudinal penetration path of chloride ions, and the Al phase dissolves preferentially due to its higher negative potential, serving as a sacrificial anode to effectively provide cathodic protection for the substrate and Fe skeleton.

Regarding practical engineering applications, the excellent corrosion resistance and sacrificial anode properties of the optimal 6Al coating make it highly promising for protecting structural steel components in harsh marine and coastal environments [36]. Furthermore, owing to the solid-state deposition characteristics of LPCS—which effectively avoids high-temperature oxidation and residual thermal stress—these Fe-Al composite coatings possess significant potential for the dimensional restoration and remanufacturing of high-value mechanical parts, such as worn pump impellers and transmission shafts [37]. Future applications could also expand into the energy and chemical sectors, serving as robust protective layers for pipeline systems where both structural continuity and electrochemical protection are critical.

## Figures and Tables

**Table 1:** Chemical composition of the 45 steel substrate (wt.%).

Elements	C	Mn	Si	P	S	Als *	Fe
Content	0.44	0.56	0.29	0.02	0.005	0.023	Bal.

* Note: The trace element Als (Acid-soluble aluminum) is a normal residual deoxidizer introduced during the industrial steelmaking process of 45 killed steel.

**Table 2:** Low-pressure cold spraying parameters.

Carrier Gas	Temperature (°C)	Pressure (MPa)	Distance (mm)	Powder Feed Rate (g/min)
Air	500~600	0.7~0.8	10~15	15

**Table 3:** Fitting parameters for the electrochemical polarization curves of composite coatings.

Coatings/wt.%	E_corr_/V	I_corr_/(A/cm^2^)	R_p_/Ω·cm^2^
2Al	−0.74077	2.564 × 10^−4^	3898.8
4Al	−0.73521	2.475 × 10^−4^	4610.1
6Al	−0.69462	2.237 × 10^−4^	5122.9
8Al	−0.70343	2.314 × 10^−4^	5069.3

**Table 4:** Fitting parameters for the equivalent circuit of the EIS data for the composite coating.

Coatings/wt.%	*R*_s_/Ω·cm^2^	*CPE*_c_/Ω^−1^·cm^−2^·s^n^	*n*_c_	*R*_c_/Ω·cm^2^	*CPE*_dl_/Ω^−1^·cm^−2^·s^n^	*n*_dl_	*R*_ct_/Ω·cm^2^
2Al	3.151	8.9665 × 10^−3^	0.57624	7.6	1.0519 × 10^−2^	0.74899	116.2
4Al	2.464	7.0682 × 10^−3^	0.61416	17.5	7.29 × 10^−3^	0.75984	136.6
6Al	2.618	1.8207 × 10^−3^	0.7361	103.6	1.5628 × 10^−3^	0.98643	291.5
8Al	2.242	2.4577 × 10^−3^	0.70745	62.8	2.6422 × 10^−3^	0.92882	257.1

## Data Availability

The original contributions presented in the study are included in the article. Further inquiries can be directed to the corresponding author.
